# Antioxidant and Anti-Inflammatory Profiles of Two Mexican *Heteropterys* Species and Their Relevance for the Treatment of Mental Diseases: *H. brachiata* (L.) DC. and *H. cotinifolia* A. Juss. (Malpighiaceae)

**DOI:** 10.3390/molecules29133053

**Published:** 2024-06-27

**Authors:** Antonio Nieto Camacho, Itzel Isaura Baca Ibarra, Maira Huerta-Reyes

**Affiliations:** 1 Instituto de Química, Universidad Nacional Autónoma de México, Ciudad Universitaria, Coyoacán 04510, Ciudad de México, Mexico; camanico2015@gmail.com; 2 Bioterio, Centro Médico Nacional Siglo XXI, Instituto Mexicano del Seguro Social, Cuauhtémoc 06720, Ciudad de México, Mexico; itzelbaca@gmail.com; 3 Unidad de Investigación Médica en Enfermedades Nefrológicas, Hospital de Especialidades “Dr. Bernardo Sepúlveda Gutiérrez”, Centro Médico Nacional Siglo XXI, Instituto Mexicano del Seguro Social, Cuauhtémoc 06720, Ciudad de México, Mexico

**Keywords:** *Heteropterys*, antioxidant, anti-inflammatory, anxiety, depression, chlorogenic acid, rutin

## Abstract

Depression and anxiety are recognized as the most common mental diseases worldwide. New approaches have considered different therapeutic targets, such as oxidative stress and the inflammation process, due to their close association with the establishment and progression of mental diseases. In the present study, we evaluated the antioxidant and anti-inflammatory activities of the methanolic extracts of the plant species *Heteropterys brachiata* and *Heteropterys cotinifolia* and their main compounds, chlorogenic acid and rutin, as potential complementary therapeutic tools for the treatment of anxiety and depression, since the antidepressant and anxiolytic activities of these methanolic extracts have been shown previously. Additionally, we also evaluated their inhibitory activity on the enzyme acetylcholinesterase (AChE). Our results revealed that both species exhibited potent antioxidant activity (>90%) through the TBARS assay, while by means of the DPPH assay, only *H. cotinifolia* exerted potent antioxidant activity (>90%); additionally, low metal chelating activity (<40%) was detected for all samples tested in the ferrozine assay. The methanolic extracts of *H. brachiata* and *H. cotinifolia* exhibited significant anti-inflammatory activities in the TPA-induced ear edema, while only *H. cotinifolia* exerted significant anti-inflammatory activities in the MPO assay (>45%) and also exhibited a higher percentage of inhibition on AChE of even twice (>80%) as high as the control in concentrations of 100 and 1000 µg/mL. Thus, the potent antioxidant and inflammatory properties and the inhibition of AChE may be involved in the antidepressant activities of the species *H. cotinifolia*, which would be positioned as a candidate for study in drug development as an alternative in the treatment of depression.

## 1. Introduction

Mental disorders comprise significant disturbances in thinking, emotional regulation, or the behavior of people and exert an impact on all aspects of life. Some estimations report that in 2019, approximately 970 million people globally were living with a mental disorder, and among these latter, anxiety and depression are the most common [1]. The recent COVID-19 pandemic further significantly increased the prevalence and burden of depression and anxiety. The estimations revealed the addition of 53.2 million new cases of depression and 76.2 million new cases of anxiety disorders globally during the year 2020 [2,3]. Despite the fact that an arsenal of antidepressant and anxiolytic drugs is available worldwide, the adverse effects, the tolerability, the complications associated with discontinuation of use, and the accessibility of the drugs themselves lead to the continuation of research for more effective, innocuous, and accessible drugs [4,5]. Consequently, a better understanding of the mechanisms of action of the current antidepressant and anxiolytic drugs, as well as of the treatments that consider different therapeutic targets, are some of the approximations that researchers have been exploring during the last decades [6]. Among these, antioxidant and anti-inflammatory activities have provided robust evidence of their role in the development of neuropsychiatric disorders, such as anxiety and depression [7]. In particular, natural products from plants have been considered potential sources of drugs for the treatment of neuropsychiatric disorders due to their potent antioxidant and anti-inflammatory properties, not only in anxiety and depression but even more so in the prevention of age-related diseases [8]. 

**Figure 1:**
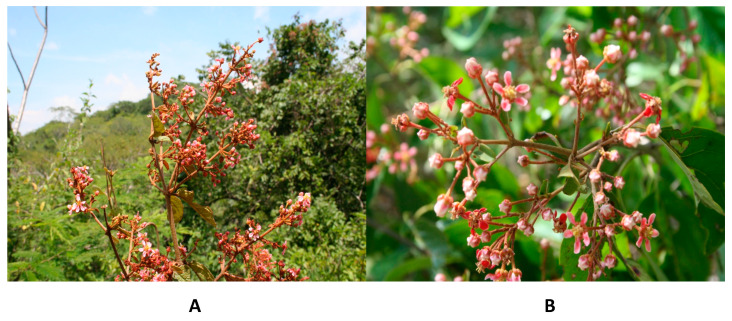
Detail of the inflorescences. (**A**) *H. brachiata*. (**B**) *H. cotinifolia.* Photo: M. Huerta-Reyes and A. Ramos-Mora.

The genus *Heteropterys*, which belongs to the Malpighiaceae family, has been studied in recent years due to its different interesting aspects. In one way, this has been because of the controversy regarding the number and origins of the lineages of this family in Mexico. Due to that, Mexico has been considered the center of origin and diversification of this family [9], in which *H. cotinifolia* has been described as endemic in Mexico while *H. brachiata* thrives from South America throughout Central America and Mexico [10]. In another manner, this is because the Malpighiaceae family has been identified as a rich source of natural products that exert activity on the Central Nervous System (CNS) in species of the genera *Galphimia*, *Byrsonima*, and *Heteropterys* [11,12,13,14]. In this respect, the species *H. brachiata* and *H. cotinifolia* are particularly relevant because of their morphological closeness (Figure 1) and due to the taxonomic controversy, since the variability of *H. cotinifolia* caused these species to be described many times and sometimes confused with *H. brachiata* [15]. Additionally, this is because they are current flora that are thriving in Mexico not only as a natural resource but also because both have been employed for the treatment of nervous disorders in Mexican Traditional Medicine in the same geographic area [16], and moreover, because their neuropharmacological properties had been described previously by our research team. In those studies, *H. cotinifolia* exhibited a dose-dependent antidepressant effect at doses ranging from 31–310 mg/kg, while no anxiolytic properties were observed. Chlorogenic acid and the flavonoid rutin were identified as the main compounds in the methanolic extract [13]. Interestingly, the same pharmacological tests (forced swimming test, elevated plus maze, and open field test) were performed in another study for the species *H. brachiata*, in which the results demonstrated that the methanolic extract of *H. brachiata* exerted a significant antidepressant effect at doses of 500 and 750 mg/kg. In contrast to *H. cotinifolia*, the methanolic extract of *H. brachiata* exerted a clear dose-dependent anxiolytic activity at 500–1500 mg/kg doses. Additionally, in this same study, *H. brachiata* exhibited anticonvulsant activity, and the extract proved to be safe since no deaths were observed in mice treated orally with 2000 mg/kg of the methanolic extract. Chlorogenic acid and the chlorogenic acid methyl ester were identified as the major compounds of the methanolic extract of *H. brachiata*, as well as less abundant terpene-type compounds [14]. Thus, from the phytochemical and neuropharmacological point of view, both species exerted properties on the CNS with different major compounds, in which the presence of the flavonoids in *H. cotinifolia* would probably be the most remarkable differentiable characteristic, and that could be useful in the taxonomic area. Furthermore, due to that, both species may constitute the basis for the development of future drugs for anxiety and depression with a natural plant origin. Based on traditional Mexican medicine, in the present study, we explored the antioxidant and anti-inflammatory activities of the methanolic extracts of the species *H. brachiata and H. cotinifolia* and their main compounds as potential complementary therapeutic tools for the treatment of anxiety and depression. Additionally, the comparison of the data between the species *H. brachiata* and *H. cotinifolia* and their main compounds was also discussed in the present contribution.

## 2. Results and Discussion 

### 2.1. TPA-Induced Ear Edema Test

**Figure 2:**
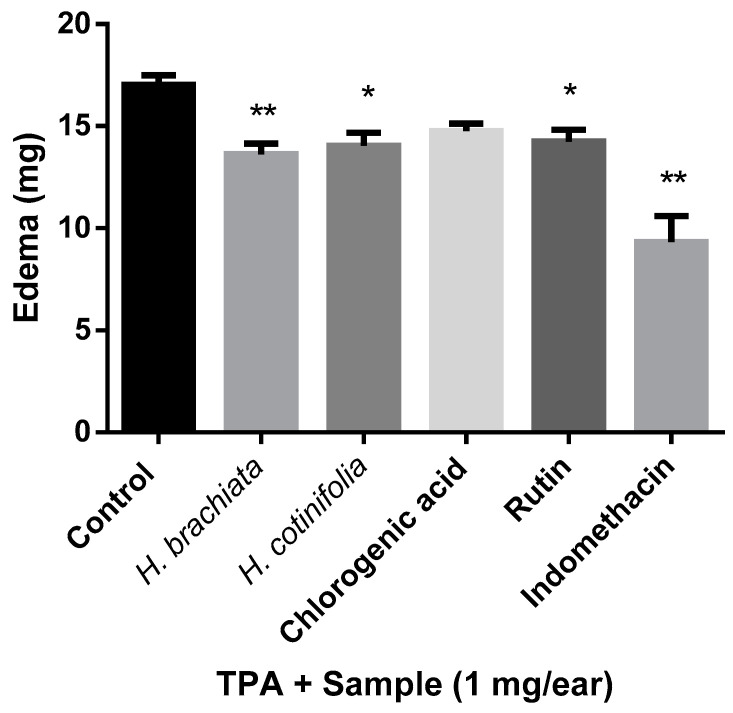
Effect of the topical administration of the methanolic extracts of *H. brachiata*, *H. cotinifolia*, and their main compounds on TPA-induced ear edema. The data represent the mean of 9 repetitions ± standard mean error (mean ± SME). All data were analyzed by ANOVA followed by a Dunnett’s test, and the values of * *p* ≤ 0.05 and ** *p* ≤ 0.01 are considered statistically different with respect to the control.

The TPA-induced ear edema test has been employed massively to evaluate the anti-inflammatory activity of plant extracts and isolated natural products due to the ear edema caused by the 12-O-tetradecanoylphorbol-13-acetate (TPA) that provokes an inflammatory response, which initially increases the levels of TNF-α. Then, the enzymes COX-2 and LOX are present together with certain other pro-inflammatory cells in order to suppress the TPA-induced inflammation. Consequently, in this model, the anti-inflammatory findings are related to COX and LOX inhibitors [17]. Our results in Figure 2 reveal that the methanolic extracts of *H. brachiata* and *H. cotinifolia* exhibited statistically significant anti-inflammatory activity when compared with the control. As has been observed in some other studies, the inflammation process in the TPA model suggests that arachidonic acid metabolism is involved. Likewise, the interaction of TPA with receptors on protein kinase C would be taking place. Additionally, as we mentioned before, inhibitors of COX and LOX, as well as corticoids and phospholipase A2, are considered active in this TPA model, and consequently, it is probably that the extracts of *H. brachiata* and *H. cotinifolia* may inhibit these mediators to inhibit TP-induced inflammation [18]. In the case of the main compounds, chlorogenic acid and rutin, only rutin exhibited significant anti-inflammatory activity when compared with Indomethacin. These results are in agreement with some other reports where chlorogenic acid demonstrated weak activity or was even considered inactive [19,20]. Thus, concerning the anti-inflammatory properties of both species of *Heteropterys* and their main compounds, only in the extracts of both species were anti-inflammatory effects detected in the TPA-induced ear edema test. 

### 2.2. Myeloperoxidase (MPO) Assay

MPO has been recognized as part of the enzymes of inflammation that are repeatedly detected in the establishment of the disease depression [21]. Furthermore, MPO has been considered a powerful marker of inflammation that is mostly expressed by neutrophils and that is actively involved in the response to oxidative stress. Thus, the protective damage of neutrophils is principally mediated by MPO, but other inflammatory markers, such as TNF-α and IL-6, are also participating here. In this manner, MPO plays an important role in the impairment of neuropsychiatric and motor behavior, in which the neurodegenerative process is affected by the increase of neutrophil infiltration in the brain. Additionally, some recent reports suggest that the inhibition of MPO, TNF-α, and IL-6 is probably involved in anxiolytic, antidepressant, and motor-behavior mechanisms [22]. 

**Figure 3:**
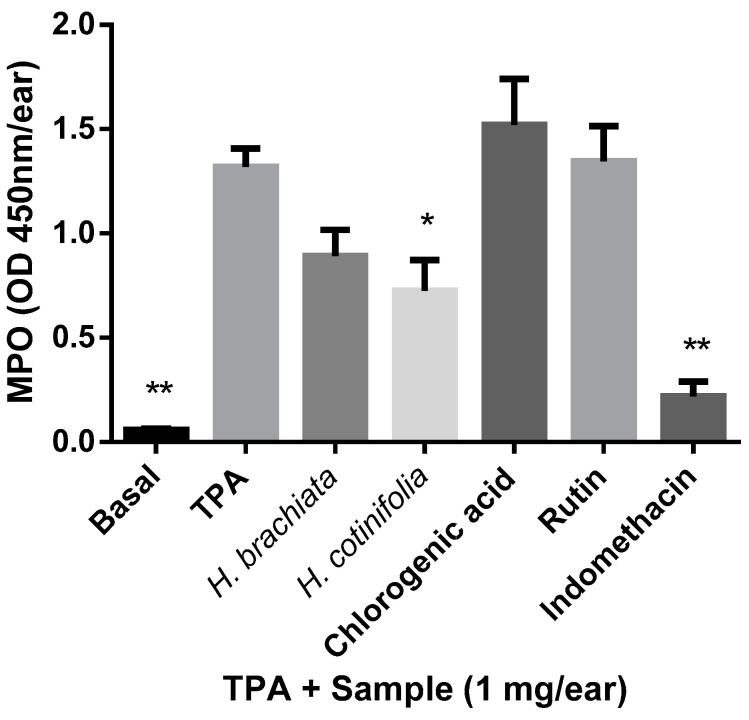
Anti-inflammatory activity of the methanolic extracts of *H. brachiata*, *H. cotinifolia*, and main compounds determined by the MPO model. The data represent the mean of 9 repetitions ± standard mean error (mean ± SME). All data were analyzed by ANOVA followed by a Dunnett’s test, and the values of * *p* ≤ 0.05 and ** *p* ≤ 0.01 are considered statistically different with respect to the TPA.

The MPO assay was employed in recent years for the evaluation of inflammatory properties in the drug discovery area of research due to the fact that the MPO enzyme content is directly related to the specific migration and cellular infiltration of neutrophils [23]. In the present study, the MPO activity was measured 4 hours after the application of the experimental samples directly from biopsies in the TPA assay. Our results (Figure 3) showed that the methanolic extract of *H. cotinifolia* was the only of the samples tested that exerted statistically significant anti-inflammatory effects on the inhibition of the edema when compared with TPA. The remaining samples, such as the methanolic extract of *H. brachiata*, and their main compounds, chlorogenic acid and rutin, did not exert significant anti-inflammatory effects in the MPO assay. In this regard, some reports in the literature that focused on other extracts of plants from species that have been used for centuries in the treatment of depression, such as a variety of *Hypericum* species, found that the anti-inflammatory effects on MPO enzyme inhibition would be related to the high oxidable content of the extracts [24]. However, the precise mechanisms involved in the anti-inflammatory effect still remain to be lucidated.

### 2.3. Inhibition of Acetylcholinesterase (AChE)

The enzyme acetylcholinesterase (AChE) is principally localized at neuromuscular junctions and cholinergic brain synapses. Its main function is related to the termination of impulse transmission at cholinergic synapses, in which, as products of the hydrolysis, acetate and choline are obtained. The AChE inhibitors block the breaking down of acetylcholine (ACh), and consequently, a longer duration of their actions is observed in the central and peripheral nervous systems. These AChE inhibitors are currently employed in the treatment of certain neurodegenerative diseases, such as Alzheimer, senile dementia, Parkinson, and ataxia [25]. Therefore, numerous screenings focused on the inhibition of the AChE have been performed for crude extracts and isolated natural products from plants in order to search for new chemical identities that would be useful in the treatment of mental illness [26,27,28]. However, not only mental age-related diseases were subjects of study; because of the inhibition of the AChE, other mental disorders, such as anxiety and depression, have also been considered. Particularly in the initial approaches, some reports suggested that the AChE inhibitors would be associated with an increase in symptoms of depression and/or anxiety in adolescents, particularly in female adolescents [29]. Contrariwise, subsequent evidence showed that the use of AChE inhibitors did not increase the symptoms of anxiety and depression in elderly adults, probably because the response to higher cholinergic stimulation is related to age-dependent effects in the CNS, and even more so because this also depends on the region of the brain responding to the specific effects [30]. In this regard, recent studies have considered that the regions of the brain that are probably involved in the complex process of anxiety and depression are not limited to the brain’s region of the hippocampus, which has been studied largely as a modulator of anxiety and depression due to the increased alteration of excitability observed in hippocampal neurons in anxiety disorder, as well as the changes in hippocampal volume, the number of synapses, and the glia-cell plasticity registered in depressive disorders. Moreover, some anxiolytic and antidepressant drugs are focused on the change in plasticity of the hippocampal neurons [31,32]. Thus, in these recent studies, other brain regions, such as the striatum and amygdala, have been considered to perhaps play a key role in anxiety and depression disorders and must be studied in depth [33].

On the other hand, the effect of herbal extracts with properties on the inhibition of AChE has also been considered in the treatment of depression and anxiety; for example, the ethanolic extract of *Salvia officinalis* showed a reduction of anxiety and an improvement in cognitive abilities after 3 days of treatment separated by a 7-day period [34]. In the case of the extract of *Melissa officinalis*, the inhibition of AChE was measured in the hippocampus [35], while the extract exhibited anxiolytic and antidepressant effects when evaluated in behavioral models in mice by inhibiting the oxidative stress and apoptotic pathways in the hippocampus and prefrontal cortex [36]. Thus, more investigations that would reveal the role of ACh inhibitors in mental diseases such as anxiety and depression are necessary, especially in the research of natural products, where there remains a pending task that remains as a possible alternative in the drug development for the treatment of anxiety and depression. 

Concerning our results, a screening was carried out for the evaluation of the inhibition of the enzyme AChE by the extracts of *H. brachiata*, *H. cotinifolia*, and their main compounds. Only the methanolic extract of *H. cotinifolia* exhibited a similar and even higher percentage of inhibition when compared with the positive control Galantamine (>35% at 0.071 µg/mL) in concentrations of 100 and 1000 µg/mL (data not shown). For this reason, we calculated the IC_50_ value of the methanolic extract of *H. cotinifolia* (Table 1). However, the IC_50_ value obtained was 97.44 ± 4.88, exhibiting non-potency in comparison with the control galantamine.

### 2.4. TBARS Assay

The TBARS assay is one of the most widely employed assays to determine oxidative stress in biological samples, especially in samples of plant products [37,38]. In the present study, FeSO_4_ was employed as an inducer since iron is able to initiate lipid peroxidation by different means, such as the formation of the Fe-O complex, the generation of the ˙OH radical, or the association with already present lipid peroxides [39]. Our results revealed the potent antioxidant effect of all of the samples, in that they exhibited ≥ 90% inhibition on the TBARS assay. Interestingly, in both cases, the extracts exhibited a very similar inhibitory effect to that of the isolated main compounds (Table 2). In the case of *H. brachiata*, the concentrations of 56.23 and 100 µg/mL exhibited 95.33 ± 0.20 and 94.41 ± 1.56% of inhibition on TBARS production, respectively, while the chlorogenic acid exerted 91.39 ± 2.81 and 95.96 ± 0.11%. For the case of *H. cotinifolia*, the extract demonstrated percentages of inhibition of TBARS production of 93.13 ± 0.49, 95.01 ± 1.00, and 95.59 ± 1.11 for the concentrations of 31.62, 56.23, and 100 µg/mL, while the flavonoid rutin exhibited 86.20 ± 1.94, 92.50 ± 0.39, and 95.72 ± 0.48, respectively. Thus, in the IC_50_ values calculated for all samples, the extract of *H. cotinifolia* showed the most potent antioxidant effect in the TBARS assay, followed by the extract of *H. brachiata*. This protector effect observed in the TBARS assay by inhibiting stress-induced oxidative damage in the brain, and particularly in depressive disorders, had also been observed for other plant extracts, such as *Nigella sativa* (Ranunculaceae) and its main active compound, thymoquinone [40]. Other plant extracts, such as garlic (*Allium sativum*) and *Melissa officinalis*, demonstrated attenuation of the depression and anxiety symptoms through their antioxidant effects in in vivo murine models due to the reduction of the MDA (malondialdehyde) levels in the TBARS assay and also because of the stimulation of the enzymatic antioxidant activity (SOD, GPx) in the brain [36,41]. Some in vitro and clinical studies showed extensively that depressive and anxiety symptoms accompanied protein and lipid peroxidation, as well as an increase in reactive oxygen species. In this manner, chemical identities that possess antioxidant activities would be useful in relieving the symptoms of depression and anxiety worldwide [36]. 

### 2.5. DPPH Assay

The DPPH (2,2-Diphenyl-1- picrylhydrazyl) assay has been used worldwide in recent decades for the determination of the antioxidant activities of foods, complex biological systems, and solid or liquid samples because of the advantages over other experimental methodologies, such as simplicity, stability, sensitivity, and inexpensiveness. The DPPH method is extensively employed for the measurement of scavenging the DPPH radical that can be displayed as antioxidant capacity and that has been employed in many screenings of a variety of plant species as extracts or fractions [42,43,44]. In the present study, our results showed, in Table 3, that the methanolic extract of *H. cotinifolia* and chlorogenic acid exerted potent antioxidant activity (≥90%) in the tested concentrations of 31.62 and 56.23 µg/mL, respectively. On comparing with other plant extracts such as *Heptaptera triquetra* fruits and their aerial parts, this species exhibited less antioxidant activity (86.19 ± 2.43 and 80.48 ± 2.72%) than *H. cotinifolia*, even at the highest concentrations tested (2000 μg ml^−1^) [45]. Furthermore, the IC_50_ value obtained for *H. cotinifolia* (IC_50 =_ 9.53 ± 0.3) results are relevant even when compared with other reports of plant extracts that are also considered antioxidants by the DPPH test and that also exhibit anxiolytic properties, as in the case of *Ocimum gratissimum* (IC_50 =_ 470 ± 28.6 µg/mL) [46].

### 2.6. Ferrozine Assay

In recent decades, oxidative stress has been associated with anxiety, depression, and other neurological disorders. Oxidative stress can be caused by reactive oxygen species, reactive nitrogen species, and unbound metal ions such as iron and copper. A direct involvement of the oxidative stress process in anxiety-like behavior was found in the hypothalamus and amygdala produced by L-buthionine-[S,R]-sulfoximine (BSO), a substance that generates oxidative stress through the inhibition of the production of glutathione (GSH) [47]. The role of copper and iron in oxidative stress and causing depression has been found recently in women suffering from postpartum depression. In addition, in patients with depression, higher copper levels and lower cortical excitability were detected, which could indicate that copper may contribute to clinical deterioration [48]. Thus, the imbalance of the metal ions contributes to the oxidative damage in a number of neurological disorders, among them anxiety and depression [49]. In this manner, in the present contribution, the metal chelating activity was measured employing ferrozine as a reagent and EDTA as a positive standard. Our results in Table 4 depict the low contribution (<40%) to the chelation activity of all samples tested when compared with the standard. On the other hand, the low chelating activity shown by the methanolic extract of *H. brachiata* and chlorogenic acid suggests that in the lipid peroxidation model induced with FeSO4, the antioxidant activity is probably mainly due to the antioxidant effect with a null participation of the chelating activity. In place, the effect of the methanolic extract of *H. cotinifolia* and rutin in the lipid peroxidation model (TBARS) may be due to a dual effect, where, on the one hand, it is due to the scavenging of free radicals, as suggested by the DPPH test; and on the other hand, the chelating activity, which, by sequestering Fe, prevents the formation of reactive oxygen species.

In this manner, in the present contribution, we explored the antioxidant and anti-inflammatory activities of the methanolic extracts of *H. brachiata* and *H. cotinifolia* and their main compounds, chlorogenic acid and rutin, due to the role that these activities play in the establishment and progression of anxiety and depression diseases. Our results reveal that the methanolic extracts of both species, *H. brachiata* and *H. cotinifolia*, possess potent antioxidant activities as determined by the TBARS and DPPH assays, that are commonly employed worldwide for the evaluation of plants. Also, the methanolic extract of *H. cotinifolia* exhibited potent inhibition on AChE, suggesting that the antioxidant and the AChE inhibition are activities that could be involved in the antidepressant activities observed in prior investigations. In this regard, *H. cotinifolia* especially could be considered a candidate for study in the drug development of antidepressant agents, and even more so in certain age-related diseases, as well as other plant extracts such as *Salvia officinalis* and *Melissa officinalis* [34,36]. Moreover, the methanolic extract of *H. cotinifolia* in the present study exhibited more potent activities than the compounds isolated, perhaps due to the complex mixtures that are present in the extracts and that involve the synergistic interaction among their components [50]. Thus, the fact that the extract demonstrated more potency than the compounds impacts long-term drug development due to the cost and time of the production of the possible phytomedicine.

Concerning the anti-inflammatory evaluation in this work, the methanolic extracts of *H. brachiata* and *H. cotinifolia* showed anti-inflammatory effects by the TPA model, and interestingly, only the methanolic extract of *H. cotinifolia* exhibited activities by the MPO model, while none of the samples tested exhibited chelating activities in the tests conducted. Since the inflammatory mechanisms are related not only to a number of mental disorders such as anxiety and depression but also to the effectiveness of some antidepressant drugs [51], our findings permit us to consider these anti-inflammatory properties and the potent antioxidant effects of other plant extracts that are widely accepted for the treatment of depression worldwide, as in the case of *Hypericum* species, in which the extract standardized is employed more than isolated compounds [52,53]. Therefore, more investigations on the specific mechanism of action through which these plant extracts of *H. brachiata* and *H. cotinifolia* cause anxiolytic and antidepressant effects are necessary in order to propose these species as a possible alternative in the treatment of anxiety and depression.

On the other hand, it cannot rule out other anti-inflammatory and antioxidant properties of drugs. Recent reports refer to the case of Histamine Type-1 and Type-2 Receptor Antagonists (H1 and H2 blockers), where H1 blockers may be related to antioxidant and anti-inflammatory activities that are also involved in the metabolic balance at the systemic level [54], while in the case of H2 receptor antagonists, antioxidant/ anti-glycation activity may be useful in neurological diseases [55]. Further research to clarify the processes involved in the antioxidant and anti-inflammatory activities of these species of the *Heteropterys* genus is necessary.

Finally, as we pointed out previously, in recent years, the Malpighiaceae family has been recognized as a potential source of phytomedicines with activity on the CNS. Moreover, a number of the most recent studies reveal that species of the Malpighiaceae family have also distinguished themselves as outstanding antioxidants, as in the case of *Malphigia emarginata* (acerola), which stands out significantly from other plants also considered potent antioxidants such as cherry, grape, strawberry, and açai. Some studies reveal that the considerable amounts of ascorbic acids present in the fresh fruits are associated with that strong antioxidant activity, as well as other molecules such as phenolic compounds, β-carotenoids, and minerals [56]. Additionally, the species *Acridocarpus orientalis* and *Banisteriopsis argyrophylla*, belonging to the Malpighiaceae family, exhibited high antioxidant activity that was related to the phenol content [57,58]. Some other species belonging to the *Byrsonima* genus also exerted strong antioxidant activities, such as *B. verbascifolia*, which possesses an abundant content of flavonoids [59], while *B. duckeana* exhibited an interesting phenol content, in which ethyl gallate was identified as the major constituent [60]. Thus, the Malpighiaceae family is positioned as an interesting source of natural products potentially useful in CNS disorders, where the antioxidant function plays a very relevant role whose study remains pending.

## 3. Materials and Methods

### 3.1. Obtaining the Methanolic Extracts of H. brachiata and H. cotinifolia

The plant samples of *H. brachiata* and *H. cotinifolia* were collected in the state of Morelos, Mexico. A voucher for each species was incorporated into the collection of the National Herbarium of Mexico’s Institute of Biology, UNAM (MEXU), with numbers 1,286,658 and 1,284,338, respectively. The extracts were obtained through the utilization of standardized methods previously reported for both species [13,61]. First, the aerial parts of each species were dried under conditions of darkness at room temperature for 15 days. Later, the dry material from each species was ground with a mill and dewaxed with *n*-hexane for 24 h. The extraction was carried out overnight by maceration with 7.5 L of methanol per kg (3×) at room temperature. Subsequently, the plant material was filtered, and the solvent was removed under reduced-pressure distillation. The methanolic extracts were stored at 4 °C until needed. The chemical composition of the methanolic extracts of both species was characterized and reported by our research group in previous manuscripts [13,14] and in the patent [61]. The main compounds of the methanolic extract of *H. brachiata* are chlorogenic acid (3.2 mg/kg) and chlorogenic acid methyl ester (60 mg/kg), while the main compounds of the methanolic extract of *H. cotinifolia* are chlorogenic acid (36.4 mg/g) and rutin (17.9 mg/g).

### 3.2. Animals

The animals were provided by the Bioterio CMN SXXI, IMSS, and their handling was performed in strict adherence to the Mexican Official Norm NOM-062-ZOO-1999 and international rules. The experimental protocol was approved by the institutional research and ethics committees (Registry number R-2023-785-095). Groups of nine CD1 male mice (weighing 25–30 g each) were used for the TPA assay. Groups of nine male Wistar rats (weighing 200–250 g each) were employed for the TBARS assay. The animals were maintained under a 12 h light/dark cycle (22 ± 1 °C) with free access to food and water.

### 3.3. TPA-Induced Ear Edema Test

Groups of nine male CD-1 mice were anesthetized with sodium pentobarbital [63 mg/Kg, intraperitoneally]. TPA 2.5 µg in ethanol (10 µL) was applied topically to the right ear, while the left ear received only ethanol. After 10 min of TPA treatment, experimental samples (methanolic extracts of *H. brachiata*, *H. cotinifolia*, chlorogenic acid, and rutin at dose of 1 mg/ear) as well as Indomethacin, the reference control, were applied dissolved in ethanol. The group of control animals received only vehicle. After 4 h, the animals were euthanized with CO_2_, and a plug of 7 mm in diameter was removed from each ear. The swelling was assessed as the difference in weight between the right- and left-ear plugs. The anti-inflammatory activity was expressed as inhibition of edema (IE): IE (%) = 100 − [B × 100/A], where A = edema induced by TPA alone and B = edema induced by TPA plus sample [62].

### 3.4. Myeloperoxidase (MPO) Assay

Tissue MPO activity was measured in biopsies taken from ears 4 h after TPA administration using an adapted method of Bradley et al. [63] and Suzuki et al. [64]. Each mouse ear biopsy was placed in 1 mL of 80 mM phosphate-buffered saline (PBS) pH 5.4 containing 0.5% hexadecyltrimethylammonium bromide (HTAB). Sample was homogenized for 30 s at 4 °C with a small sample laboratory Tissue Tearor Homogenizer (OMNI International, model 125). The homogenate was freeze-thawed at room temperature (3×), sonicated for 20 s, and then centrifuged at 12,000 rpm for 15 min at 4 °C. Quadruplicates of 10 μL of the resulting supernatant were poured into a 96-well microplate, and 180 μL of 80 mM PBS (pH 5.4) without HTAB were added. Microplate was heated to 37 °C, and then 20 μL of 0.017% hydrogen peroxide were added to each well. For the MPO activity, 20 μL of 18.4 mM 3,3′,5,5′, tetramethylbenzidine in 50% aqueous dimethylformamide were added to start the reaction. Microtiter plates were incubated at 37 °C for 5 min. Reaction was stopped with 20 μL of 2 M H_2_SO_4_. MPO enzyme activity was assessed calorimetrically using a BioTek Microplate Reader (SYNERGY HT) at an absorbance wavelength of 405 nm. MPO activity was expressed as optical density (OD) per biopsy.

### 3.5. Inhibition of Acetylcholinesterase (AChE)

An enzyme solution (50 μL, 0.195 U mL^−1^) will be added to a solution (50 μL) of the extract to be evaluated in 2% MeCN in PBS (11.3 mM, pH 7.4) and incubated for 30 min at 25 °C. The reaction will be initiated by adding 100 μL of substrate solution containing 5,5′-dithiobis (2-nitrobenzoic acid) (0.20 mM, DTNB) and 0.24 mM acetylthiocholine iodide (ATChI) in PBS (15.85 mM, pH 7.5). The formation of colorful products was measured on a Synergy HT Bio Tek microplate reader at 412 nm after 5 min. Galantamine will be used as a positive control [65].

### 3.6. TBARS Assay

Lipid peroxidation was measured by TBARS assay using rat brain homogenates, where their supernatants (375 mL) were mixed with 50 mL of 10 µM EDTA and 25 µL of experimental samples (methanolic extracts of *H. brachiata*, *H. cotinifolia*, chlorogenic acid, and rutin) or butylated hydroxytoluene (BHT) that was employed as reference standard, and these were incubated for 30 min at 37 °C. Fifty µL of 100 µM FeSO_4_ were added to induce lipid peroxidation, and the mixture was incubated at 37 °C for 60 min. Subsequently, 500 mL of thiobarbituric acid (TBA) solution (0.5% TBA in 0.05 N NaOH and 30% trichloroacetic acid at a 1:1 proportion) were added. The mixture was centrifuged at 12,879× *g* for 5 min and incubated for 30 min at 80 °C. Absorbance of the supernatant was measured at 540 nm in a Bio-Tek Microplate reader ELx808. The concentration of TBARS was calculated by interpolation in a standard curve of tetramethoxypropane 1,1,3,3 (TMP) as a precursor of malondialdehyde (MDA) [39,66]. Results were expressed as nmoles of TBARS per mg of protein. The inhibition ratio [IR (%)] was calculated using the following formula: IR = (Abs_control_ − Abs_sample_) × 100/Abs_control_. 

### 3.7. DPPH Assay

The antioxidant activity of the methanolic extracts of *H. brachiata*, *H. cotinifolia*, and their main compounds was carried out by mixing 50 µL of the samples with 150 µL of the ethanolic solution of DPPH (final concentration, 100 µM). After incubation at 37 °C for 30 min in the dark, the absorbance of DPPH solutions was measured at 515 nm in a microplate reader Synergy HT (BioTek Instruments, Winooski, VT, USA). Scavenging capacity (%) is calculated as [(Absc_ontrol_ − Abs_sample_)/(Abs_control_) × 100]; quercetin was used as standard [67].

### 3.8. Ferrozine Assay

The metal chelating activity was assessed by mixing 0.16 mL of experimental samples (at concentration of 1 µg/mL) with 0.5 mM FeSO_4_ (0.02 mL) and 8 mM ferrozine (0.02 mL). After incubation for 10 min at room temperature, absorbance was measured at 562 nm in a Synergy HT microplate reader (BioTek Instruments, Winooski, VT, USA). Metal chelating activity (%) was calculated as [(A_562_ of control − A_562_ of sample/A_562_ of control] × 100. EDTA was employed as standard [68].

## 4. Conclusions

Our findings showed that the methanolic extract of the species *H. cotinifolia* exhibited the most potent antioxidant activity, anti-inflammatory properties, and AChE inhibition effects among all samples tested. Therefore, this extract could be viewed as a potential candidate for drug development, serving as an alternative treatment for depression. This is particularly due to its cost-effectiveness and low production costs. Additionally, conducting further in vivo biological studies would be a beneficial next step.

## Figures and Tables

**Table 1:** Dose-response curve concentration and IC_50_ values of the methanolic extract of *H. cotinifolia* on AChE inhibition.

Sample	Concentration (µg/mL)	O.D. (412 nm)	Inhibition (%)	IC_50_ (µg/mL)
*H. cotinifolia* ^a^	1	0.366 ± 0.020	9.16 ± 4.59	97.44 ± 4.88
	3.16	0.356 ± 0.017	11.65 ± 4.00	
	10	0.322 ± 0.014 **	19.99 ± 3.32 **	
	31.62	0.275 ± 0.013 **	31.69 ± 3.01 **	
	100	0.197 ± 0.008 **	51.14 ± 1.89 **	
	316.23	0.081 ± 0.007 **	79.94 ± 1.89 **	
	1000	0.012 ± 0.006 **	96.97 ± 1.52 **	
Galantamine ^b^	0.00037	0.374 ± 0.014 *	18.77 ± 3.30 *	0.09 ± 0.03
	0.0037	0.343 ± 0.022 **	25.26 ± 4.08 **	
	0.037	0.256 ± 0.022 **	43.94 ± 4.84 **	
	0.37	0.120 ± 0.022 **	74.12 ± 4.55 **	
	3.68	0.026 ± 0.007 **	94.43 ± 1.54 **	

The data represent the mean of 3 repetitions ± standard mean error (mean ± SME). All data were analyzed by ANOVA followed by a Dunnett’s test, and the values of * *p* ≤ 0.05 and ** *p* ≤ 0.01 are considered statistically different with respect to the control. (^a^ 0.402 ± 0.003, ^b^ 0.461 ± 0.021).

**Table 2:** Concentration response curve and IC_50_ values of *H. brachiata*, *H. cotinifolia*, and their main compounds on the TBARS assay.

Sample ^a^	Concentration (µg/mL)	TBARS (nmol/mg prot)	Inhibition (%)	IC_50_ (µg/mL)
*H. brachiata* ^b^	5.62	6.15 ± 0.57 *	21.60 ± 4.59 *	13.80 ± 1.04
	10	4.89 ± 0.55 **	37.77 ± 5.43 **	
	17.78	3.02 ± 0.44 **	61.81 ± 5.52 **	
	31.62	0.92 ± 0.17 **	88.35 ± 2.48 **	
	56.23	0.67 ± 0.03 **	95.33 ± 0.20 **	
	100	0.43 ± 0.05 **	94.41 ± 1.56 **	
*H. cotinifolia* ^c^	5.62	5.67 ± 0.45 **	27.29 ± 8.76 **	11.52 ± 0.46
	10	4.51 ± 0.36 **	42.31 ± 4.55 **	
	17.78	1.69 ± 0.27 **	78.63 ± 3.58 **	
	31.62	0.54 ± 0.05 **	93.13 ± 0.49 **	
	56.23	0.39 ± 0.05 **	95.01 ± 1.00 **	
	100	0.35 ± 0.06 **	95.59 ± 1.11 **	
Chlorogenic acid ^d^	5.62	7.76 ± 0.40	−0.10 ± 3.87	22.05 ± 1.55
	10	6.96 ± 0.44	10.32 ± 3.72	
	17.78	4.74 ± 0.41 **	39.12 ± 4.31 **	
	31.62	1.83 ± 0.23 **	76.61 ± 3.44 **	
	56.23	0.66 ± 0.11 **	91.39 ± 2.81 **	
	100	0.31 ± 0.01 **	95.96 ± 0.11 **	
Rutin ^e^	5.62	7.24 ± 0.40	7.13 ± 4.35	14.20 ± 1.27
	10	5.41 ± 0.46 **	28.34 ± 8.50 **	
	17.78	2.44 ± 0.31 **	66.24 ± 8.46 **	
	31.62	1.03 ± 0.10 **	86.20 ± 1.94 **	
	56.23	0.59 ± 0.05 **	92.50 ± 0.39 **	
	100	0.35 ± 0.02 **	95.72 ± 0.48 **	
BHT ^f^	0.17	5.56 ± 0.29 *	23.92 ± 2.69 **	0.27 ± 0.10
	0.22	4.46 ± 0.28 **	37.14 ± 7.44 **	
	0.29	3.23 ± 0.57 **	53.59 ± 8.93 **	
	0.39	1.32 ± 0.49 **	81.59 ± 6.89 **	
	0.52	0.49 ± 0.08 **	93.16 ± 1.16 **	
	0.70	0.53 ± 0.04 **	95.08 ± 0.68 **	

The data represent the mean of six animals ± standard mean error (mean ± SME). All data were analyzed by ANOVA followed by a Dunett’s test, and the values of * *p* ≤ 0.05 and ** *p* ≤ 0.01 are considered statistically different with respect to the control. (Basal ^a^ 0.600 ± 0.252. Control ^b^ 9.108 ± 0.123, ^c^ 9.319 ± 0.293, ^d^ 9.701 ± 0.467, ^e^ 9.786 ± 0.268, ^f^ 7.384 ± 0.630).

**Table 3:** IC_50_ values of *H. brachiata*, *H. cotinifolia*, and their main compounds in the DPPH assay.

Sample	Concentration (µg/mL)	O.D. (515 nm)	DPPH Reduction (%)	IC_50_ (µg/mL)
*H. brachiata*	3.16	0.614 ± 0.003 **	9.74 ± 1.25 **	25.65 ± 0.12
	5.62	0.566 ± 0.007 **	16.77 ± 0.80 **	
	10	0.521 ± 0.002 **	23.52 ± 1.20 **	
	17.78	0.428 ± 0.006 **	37.15 ± 0.33 **	
	31.62	0.281 ± 0.005 **	58.75 ± 0.17 **	
	56.23	0.091 ± 0.002 **	86.65 ± 0.05 **	
*H. cotinifolia*	3.16	0.531 ± 0.012 **	18.97 ± 0.66 **	9.53 ± 0.3
	5.62	0.447 ± 0.008 **	31.87 ± 0.13 **	
	10	0.320 ± 0.009 **	51.15 ± 0.48 **	
	17.78	0.111 ± 0.005 **	83.04 ± 0.53 **	
	31.62	0.057 ± 0.001 **	91.22 ± 0.21 **	
	56.23	0.055 ± 0.001 **	91.63 ± 0.13 **	
Chlorogenic acid	3.16	0.585 ± 0.004 **	10.69 ± 1.47	16.13 ± 0.22
	5.62	0.535 ± 0.005 **	18.47 ± 1.09	
	10	0.451 ± 0.002 **	31.12 ± 1.22	
	17.78	0.303 ± 0.005 **	53.76 ± 0.88	
	31.62	0.094 ± 0.003 **	85.67 ± 0.13	
	56.23	0.044 ± 0.003 **	93.25 ± 0.33	
Rutin	3.16	0.647 ± 0.010	1.29 ± 0.84	8.2 ± 0.15
	5.62	0.620 ± 0.002 **	5.40 ± 1.46 **	
	10	0.528 ± 0.002 **	19.35 ± 1.39 **	
	17.78	0.271 ± 0.003 **	58.66 ± 0.45 **	
	31.62	0.074 ± 0.001 **	88.65 ± 0.05 **	
	56.23	0.069 ± 0.002 **	89.48 ± 0.07 **	
Quercetin	1.07	0.560 ± 0.004 **	17.03 ± 0.79 **	3.67 ± 0.13
	1.90	0.491 ± 0.007 **	27.14 ± 1.24 **	
	3.38	0.363 ± 0.008 **	46.21 ± 1.29 **	
	6.01	0.167 ± 0.016 **	75.17 ± 2.40 **	
	10.69	0.041 ± 0.001 **	93.87 ± 0.19 **	

The data represent the mean of 3 repetitions ± standard mean error (mean ± SME). All data were analyzed by ANOVA followed by a Dunnett’s test, and the values of * *p* ≤ 0.05 and ** *p* ≤ 0.01 are considered statistically different with respect to the control.

**Table 4:** Metal chelating activity of *H. brachiata*, *H. cotinifolia*, and their main compounds.

Sample	O.D. (562 nm)	Chelation (%)
Control	0.785 ± 0.013	
*H. brachiata*	0.729 ± 0.030	7.27 ± 2.83
*H. cotinifolia*	0.697 ± 0.012 *	11.21 ± 0.93 *
Chlorogenic acid	0.850 ± 0.015	−8.28 ± 1.09 (NO ACTIVITY)
Rutin	0.505 ± 0.014 **	35.68 ± 0.99 **
Quercetin	0.812 ± 0.020	−3.44 ± 3.97 (NO ACTIVITY)
EDTA	0.002 ± 0.001 **	99.73 ± 0.09 **

The data represent the mean of three repetitions ± standard mean error (mean ± SME). All data were analyzed by ANOVA followed by a Dunett’s test, and the values of * *p* ≤ 0.05 and ** *p* ≤ 0.01 are considered statistically different with respect to the control.

## Data Availability

Data are contained within the article.

## Abbreviations

AChE	Acetylcholinesterase Enzyme
TBARS	Thiobarbituric Acid Reactive Substances
DPPH	(2,2-Diphenyl-1-picrylhydrazyl)
TPA	12-O-tetradecanoylphorbol-13-acetate
MPO	Myeloperoxidase
CNS	Central Nervous System
TNF-α	Tumor Necrosis Factor-alpha
COX-2	Cyclooxygenase-2
IL-6	Interleukin-6
MDA	Malondialdehyde
GSH	Glutathione
OD	Optical Density
HTAB	Hexadecyltrimethylammonium Bromide
TBA	Thiobarbituric Acid
TMP	Tetramethoxypropane 1,1,3,3
SOD	Superoxide Dismutase
GPx	Glutathione Peroxidase
